# Proteomic Characterization of Microbiota-Derived Extracellular Vesicle-Enriched Fractions Detected in Red Yeast Rice

**DOI:** 10.3390/biom16101425

**Published:** 2026-10-01

**Authors:** Wei Qi, Xuan-Xi Peng, Pei-Yan Song, San-Tong Xiao, Wen-Zhong Hu

**Affiliations:** School of Life Science, Zhuhai College of Science and Technology, Zhuhai 519041, China; pengxuanxicc@stu.zcst.edu.cn (X.-X.P.); songpeiyan@stu.zcst.edu.cn (P.-Y.S.); xiaosantong@stu.zcst.edu.cn (S.-T.X.)

**Keywords:** extracellular vesicles-enriched fractions, proteomic, red yeast rice, microbiota-derived

## Abstract

Fermented foods confer health benefits partially through microbial extracellular vesicles (EVs); however, complex fermented food-derived EVs remain understudied. In this research, we isolated and characterized microbiota-derived EV-enriched fractions (REVs) from 24 red yeast rice (RYR) samples obtained from three Chinese regions. The morphological analysis showed that the EV-enriched fractions exhibited a typical spherical, lipid bilayer structure. Proteomic profiling identified 2376 proteins, including 273 microbiota-derived proteins primarily originating from *Monascus*, *Pectobacterium*, and *Erwinia species*, with 143 proteins showing differential expression among regions. Functional analyses revealed significant enrichment in transport, glycolysis, translation, RNA degradation, and amino acid metabolism. Furthermore, we identified candidate region-associated proteins: rpsK and atpD in the FJ region; ypt-1, ALA1, and MIP11 in the GX region; and trxA, BAB1_1794, pstS, ugpB, tal, and glpK in the ZJ region. As the first comprehensive proteomic characterization of REVs, this study provides a basis for future studies on region-associated protein signatures and the potential application of fermented food-derived EVs in functional foods.

## 1. Introduction

Understanding the bioactive components in human diets is essential for gaining insights into how nutrition affects health, contributes to disease development, and informs the development of functional foods and nutraceuticals [1]. Fermented products are recognized for their exceptional nutritional content and unique biochemical properties, making them important components of a health-conscious diet [2]. Research on fermented matrices has focused primarily on their macro- and micronutrients, such as dietary fiber, polyphenols, polysaccharides, and the live microbial communities themselves. These microorganisms, including various fungi, yeasts, and bacteria, interact dynamically with the human immune system to support overall health. Additionally, these components promote intestinal epithelial regeneration, beneficially modify the gut microbiome, and exert protective effects against certain diseases, such as liver steatosis and colitis, often through mechanisms involving specific microRNAs and lipid pathways [3].

Researchers are increasingly looking beyond live cells to understand microbe–host interactions. Extracellular vesicles (EVs), which are nonreplicating, lipid bilayer-bound nanoparticles that typically range from 30 to 1000 nanometers in diameter, are produced and secreted across all biological domains [4]. By serving as natural delivery systems, EVs internalize and transport a variety of bioactive molecules, including metabolites, lipids, nucleic acids, and proteins. Their lipid envelope provides notable structural stability, protecting these molecular payloads from enzymatic degradation in challenging physiological environments. Upon reaching target tissues, EVs are taken up by cells, thereby initiating specific biological responses [5]. Recent evidence has further indicated that EVs may mediate interspecies and even cross-kingdom communication through the delivery of functional molecular cargoes [6]. Notably, research has shown that EVs are important mediators of intercellular communication, immune regulation, the stress response, and the maintenance of intestinal homeostasis [7,8,9,10,11,12,13,14].

To our knowledge, the presence of extracellular vesicles (EVs) has been documented in various fermented staple foods, including kombucha [15], dairy products [16] and kefir [17]. As a result, individuals are continually exposed to these diverse vesicular populations through the gastrointestinal tract throughout their lifespan [8,18,19]. This ongoing exposure leads to the hypothesis that the health-promoting effects commonly attributed to dietary probiotics and supplements may be largely mediated by their secreted EVs rather than solely by live microorganisms. Emerging research on probiotic yeasts and lactic acid bacteria supports this association [11,20,21]. Additionally, the use of these vesicles may reduce the risk of infection associated with the use of whole microbial cells [22]. Consequently, recognizing EVs derived from fermented foods as novel bioactive dietary components is important. A comprehensive assessment of their therapeutic and biotechnological potential will contribute to a deeper understanding of food fermentation ecosystems [23].

Red yeast rice (RYR), also referred to as red fermented rice, red leaven, or *Hongqu*, is a traditional fermented product that has been consumed in East Asia for centuries [24]. It is commonly used as a food colorant, flavor enhancer, and preservative in Chinese, Korean, and Japanese culinary practices. Additionally, RYR has gained international recognition as a dietary supplement and traditional medicine, particularly in the management of hyperlipidemia [25,26]. However, further research is necessary to fully understand the molecular mechanisms underlying the health benefits of RYR. The complex composition of the RYR microbiota poses challenges for conducting detailed analyses of microbe–host interactions. Additionally, although extracellular vesicles from other sources have been examined, the mechanisms through which RYR microbiota-derived extracellular vesicle-enriched fractions (REVs) influence recipient cells are currently poorly understood. This knowledge gap is due mainly to limited information concerning the protein composition and biological contents of these specific vesicles.

This study aims to investigate the microbial functional proteins and microbiota-associated vesicle proteins present in red yeast rice, with an emphasis on exploring their potential functions. Using a combination of biophysical and biochemical techniques, we first identified vesicle-like particles and obtained EV-enriched fractions from RYR samples. We subsequently analyzed 24 REVs isolated from RYR collected from three different geographic regions using a liquid chromatography system coupled with a high-resolution Orbitrap Elite tandem mass spectrometer (MS/MS). Proteins were identified using FragPipe v23.1 software, and bioinformatics analyses were conducted to categorize their subcellular localizations and associated biological processes, with the goal of exploring the region-specific characteristics of the EV-enriched fractions. Additionally, the taxonomic and functional compositions of the associated microbial communities were examined. Our results also suggest that the EV-enriched fractions from RYR may contain vesicle-associated components derived from both bacterial and fungal sources. To the best of our knowledge, this study is the first to characterize the proteome of microbiota-derived EV-enriched fractions from red yeast rice. These results provide a foundation for further research into the potential roles of these proteins in host–microbe interactions and their applications in functional food development.

## 2. Materials and Methods

### 2.1. Materials

All 24 autochthonous red yeast rice samples were collected from local traditional producers in three representative provinces in China: Fujian, Guangxi, and Zhejiang. Each sample was obtained from a different producer and represented an independent sample. Therefore, in this study, one red yeast rice sample from one producer was defined as one biological replicate and as the experimental unit for statistical analysis. Following collection, all the samples were transported to the laboratory using ice packs and stored at −80 °C until subsequent analysis. Samples obtained from different producers were never pooled or combined at any stage of the study. The geographical origin information of the samples is summarized in Table S1.

### 2.2. Extraction of Extracellular Vesicles from Red Yeast Rice Samples

The red yeast rice samples were diluted with phosphate-buffered saline (PBS) to a final volume of 5 mL and subsequently filtered through a 0.22 μm membrane filter (Millipore, MA, USA). The suspension from each individual sample was then subjected to EV isolation using the EXODUS method [27] with alternative vacuum actuation under the specified conditions of −20 kPa with a 10 s cycle time. After isolation, each subfraction was further eluted twice with PBS using the EXODUS system. Finally, the three fractions from the same individual sample were pooled and resuspended in 200 μL of PBS and stored at −80 °C. The resulting preparation was considered an EV-enriched fraction for future analysis.

### 2.3. TEM Imaging, NTA Measurements, and Protein Quantification of REVs

The morphology of REVs was examined using negative staining. Ten microliters of the purified vesicle suspension was applied to glow-discharged, carbon-coated formvar 200-mesh copper grids and incubated for 30 s. Following rapid blotting, the grids were stained with a commercial uranyl acetate solution from Delta Microscopies (Mauressac, France) for 30 s. Imaging was conducted using a transmission electron microscope (JEOL, Tokyo, Japan) [15].

Size distribution and concentration assessments were performed using nanoparticle tracking analysis (NTA) with a ZetaView PMX 120 instrument (Particle Metrix, Meerbusch, Germany) utilizing ZetaView 8.05.14 SP7 software. A total of 24 EV-enriched fractions, each independently isolated from one individual red yeast rice sample, were analyzed. Measurements were conducted at 25 °C in phosphate-buffered saline (PBS) supplemented with 8.1 mM sodium phosphate, 136.9 mM sodium chloride, 2.68 mM potassium chloride, and 1.47 mM potassium phosphate (pH 7.0), which was subsequently filtered through a 0.22 μm filter (Lonza, Basel, Switzerland). Each sample was recorded in triplicate for 60 s with a camera level of 12 and a threshold setting of 2 [28]. Samples were analyzed using a 488 nm laser and scatter filter across 11 positions during software-based analysis. The image analysis settings were minimum brightness 30, minimum area 10, and maximum area 1000.

The protein concentration of the EV-enriched fractions was measured using a Qubit Protein Assay Kit (Thermo Fisher Scientific, Bremen, Germany, Cat. No. Q33211) according to the manufacturer’s instructions.

### 2.4. Protein Digestion

The protein solutions were initially diluted in 50 mM ammonium bicarbonate (ABC). The proteins were reduced using 10 mM dithiothreitol (DTT; Merck, Darmstadt, Germany) at 56 °C for 45 min. The cysteine residues were subsequently alkylated with 40 mM iodoacetamide (IAM; Sigma, St. Louis, MO, USA) at 25 °C for 30 min. The samples were then subjected to enzymatic digestion with sequencing-grade modified porcine trypsin (Promega, Madison, WI, USA) at 37 °C for 16 h. Following digestion, the peptides were desalted, dried via vacuum centrifugation, and stored at −80 °C until further analysis.

### 2.5. LC-MS/MS Analysis

Tryptic peptide samples were analyzed using nanoflow LC-MS/MS on a Q Exactive HF-X Orbitrap mass spectrometer (Thermo Fisher Scientific, Bremen, Germany) coupled with an EASY nLC 1200 ultrahigh-pressure liquid chromatography system (Thermo Fisher Scientific, Bremen, Germany) with a nanoelectrospray ion source. Approximately 500 ng of peptide was loaded onto a 25 cm analytical column (150 μm inner diameter) packed with ReproSil-Pur C18-AQ 1.9 μm silica beads (Beijing Qinglian Biotech Co., Ltd., Beijing, China). Peptides were separated using a linear gradient: from 8% to 12% solvent B over 5 min, 12% to 30% solvent B over 33 min, and then increased to 40% solvent B over 7 min, followed by a 15 min wash at 95% solvent B. The total run time was approximately 60 min. The column temperature was maintained at 60 °C using a custom-built oven. The solvent conditions consisted of solvent A (0.1% formic acid in water) and solvent B (80% acetonitrile with 0.1% formic acid in water). The flow rate was set at 600 nL/min. The mass spectrometry analysis was performed in data-dependent “top−40” mode. MS spectra were acquired with the Orbitrap analyzer at a resolution of 120,000 over the 350–1500 *m*/*z* range, with an AGC target of 3 × 10^6^ and a maximum injection time of 80 ms. The most intense ions identified in the full scan were isolated with a width of 1.6 m/z for subsequent fragmentation. Higher-energy collisional dissociation (HCD) was performed with a normalized collision energy of 27. MS/MS spectra were collected in the Orbitrap at a resolution of 15,000, with an AGC target of 5 × 10^4^ and a maximum injection time of 45 ms. Precursor ions were dynamically excluded for 16 s to prevent repeated analysis of the same ions.

### 2.6. Protein Identification and Quantification

Raw LC-MS/MS data were processed using the FragPipe suite. MS/MS spectra were searched against the UniProtKB microorganism proteome database, including both Swiss-Prot and TrEMBL microorganism reference protein sequences. The search parameters were as follows: full tryptic specificity, a maximum of two missed cleavages, a minimum peptide length of 6 amino acids, fixed carbamidomethylation of cysteine residues (+57.02146 Da), and variable oxidation of methionine residues (+15.99492 Da). Percolator was used to control the quality of peptide spectral matches, with a false discovery rate (FDR) of less than 1% at the peptide level. The peptides were then assembled into protein identifications, which were further filtered to achieve a final protein-level FDR of 1%. For quantification, unique and razor peptides were used to estimate protein abundance, and protein abundance tables were generated for downstream analysis. Only microbial peptides and proteins were included in subsequent analyses, with features not assigned to bacterial, archaeal, or fungal taxa manually excluded. Protein inference and taxonomic assignment were performed using FragPipe with the built-in Philosopher workflow. Philosopher applies a peptide-centered protein inference strategy in which proteins supported by the same set of identified peptides, but lacking unique peptides that distinguish them, are grouped as indistinguishable proteins. One representative (master) protein is reported in the Protein ID, whereas the remaining ambiguous members are listed in the Indistinguishable Proteins. For downstream analyses, including taxonomic summarization, only the representative (master) protein was used for reporting and quantification. Thus, shared-peptide signals were counted once at the protein-group level and were not repeatedly assigned to all indistinguishable proteins within the same group.

### 2.7. Bioinformatics Analysis

Gene Ontology (GO) and InterPro (IPR) annotations were determined based on protein sequence analysis, and protein family and pathway analyses were conducted using the Clusters of Orthologous Groups (COG) and Kyoto Encyclopedia of Genes and Genomes (KEGG) databases. GO term and KEGG pathway enrichment analyses were performed by over-representation analysis using a hypergeometric test. For enrichment analyses of differentially expressed proteins (all, upregulated, and downregulated proteins), all proteins identified in the corresponding proteomic dataset were used as the background set. For enrichment analyses of the complete protein set at the sample level, the full annotation database was used as the background set. Multiple-testing correction was performed using the Benjamini–Hochberg method, and both nominal *p* values and FDR-adjusted *p* values were recorded.

### 2.8. Statistical Analyses

Raw protein abundance data were median-normalized using the R package preprocessCore (v1.66.0) to reduce systematic experimental variation. For principal component analysis (PCA) and correlation heatmap analysis, proteins containing zero values were removed from the normalized dataset to generate a complete matrix without missing values. For differentially expressed proteins (DEPs) analysis, the normalized protein abundance data was imported into Perseus for missing-value imputation using a downshifted normal distribution (down shift = 1.8, width = 0.3). Pairwise comparisons between regions were conducted using a two-sample Student’s *t*-test. Raw *p* values were further adjusted for multiple testing using the Benjamini–Hochberg method to calculate false discovery rate (FDR) values. In this study, proteins with nominal *p* < 0.05 and fold change (FC) > 1.2 or <1/1.2 were defined as candidate DEPs for exploratory screening subsequent functional analyses. Benjamini–Hochberg-adjusted false discovery rate (FDR) values were additionally calculated for all quantified proteins and are reported in the Supplementary Materials. As multiple-testing correction substantially reduced the number of statistically supported signals, DEP-based regional comparisons and enrichment results should be interpreted as exploratory rather than confirmatory. Data visualization was conducted using R software (version 4.3.2), GraphPad Prism (version 8.0.2), and the Bioladder-Online Bioinformatics Analysis and Visualization Cloud Platform (https://www.bioladder.cn/).

## 3. Results and Discussion

### 3.1. Characteristics of Microbiota-Derived EV-Enriched Fractions from Red Yeast Rice

**Figure 1:**
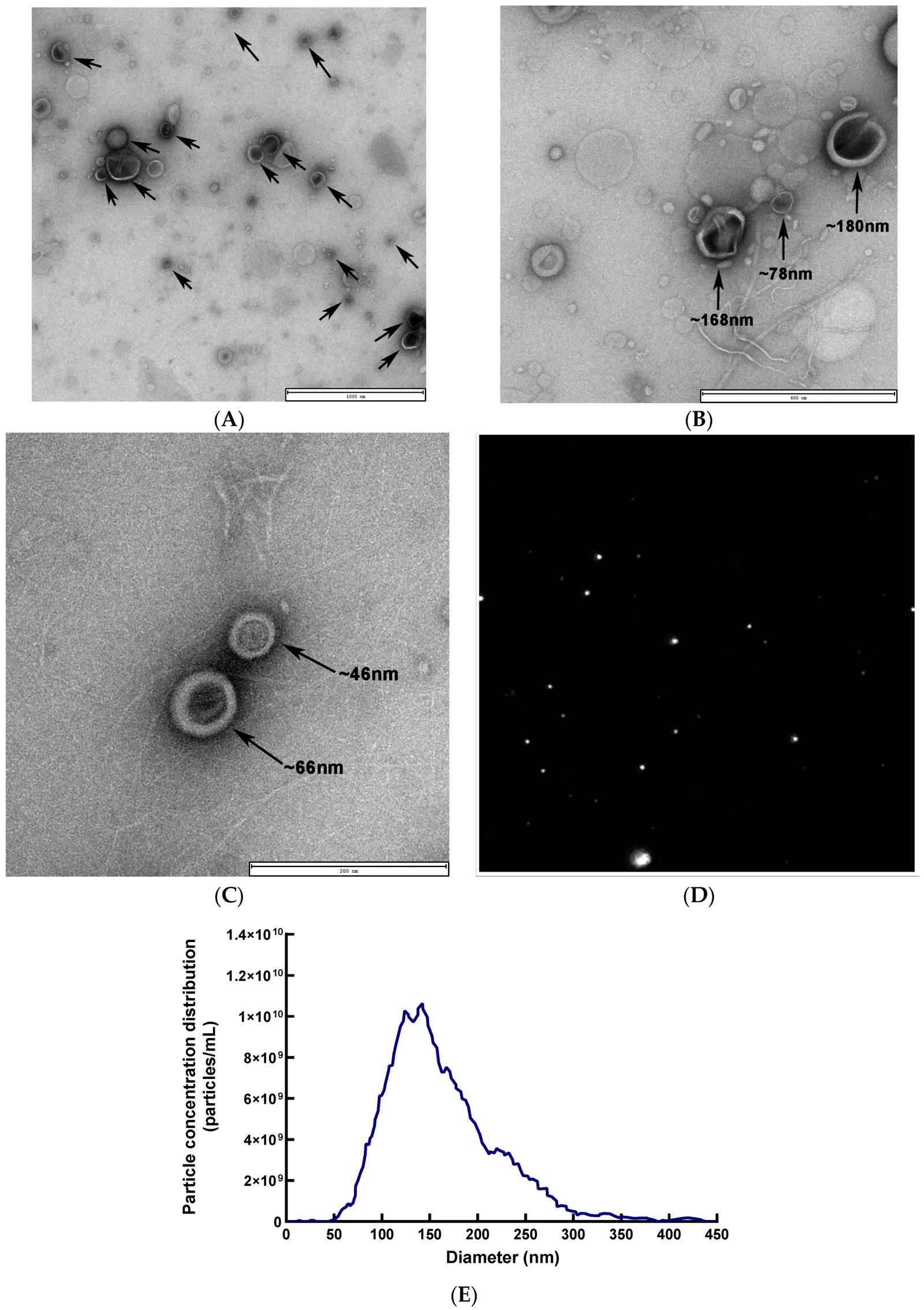
Morphology and particle-size distribution of microbiota-derived EV-enriched fractions from red yeast rice. (**A**–**C**) Transmission electron microscopy (TEM) images of the vesicle-enriched fraction isolated from red yeast rice. Multiple fields of view and magnifications reveal nanoscale particles with a vesicle-like morphology, including round and cup-shaped membrane-bound structures. Scale bars: (**A**) 1000 nm; (**B**) 600 nm; and (**C**) 200 nm. (**D**) Image obtained from a nanoparticle tracking analysis (NTA) video. (**E**) Representative NTA size distribution curve of the EV-enriched fraction isolated from red yeast rice (sample 1). Analysis was performed using a ZetaView instrument across 11 measurement positions, with one position excluded during analysis. The original particle concentration was 2.3 × 10^11^ particles/mL, with a mean particle size of 153.0 nm, a median diameter (X50) of 140.6 nm, X10 and X90 values of 90.2 and 227.6 nm, respectively. The summary statistics for all 24 samples are shown in Table 1.

The microbiota-derived EV-enriched fractions isolated from red yeast rice were characterized by transmission electron microscopy (TEM), nanoparticle tracking analysis (NTA), and protein quantification (Figure 1 and Table 1). TEM revealed numerous nanoscale vesicle-like particles in the isolated fraction. Examinations across multiple fields of view and at high magnification revealed predominantly round or cup-shaped, membrane-enclosed structures, indicating the presence of nanoscale vesicle-like particles in the sample (Figure 1A–C) [21,29].

NTA was used to determine the particle-size distribution and concentration of the EV-enriched fractions isolated from red yeast rice. In a total of 24 independently prepared samples, the particle concentrations ranged from 9.4 × 10^10^ to 1.3 × 10^12^ particles/mL, with an overall mean of 4.60 ± 3.44 × 10^11^ particles/mL. The average particle size ranged from 110.8 to 180.5 nm, with a mean of 135.5 ± 15.5 nm (Table 1). A representative NTA size distribution curve for sample 1 is shown in Figure 1E, displaying a prominent peak within the nanoscale range (mean size: 153.0 nm; median size (X50): 140.6 nm; X10: 90.2 nm; X90: 227.6 nm). The original ZetaView NTA report, the 11-position measurement summary, and the raw output file for the representative sample 1 are provided in the Supplementary Materials (Data S1–S3). While this sample exhibited a primarily single-peak distribution, the broader dataset demonstrated variability in mean particle size across samples, reflecting the heterogeneity typical of complex fermentation-derived materials. The particle size range observed here is broadly consistent with that previously reported for food-derived vesicles (50–300 nm) [30], yeast (20–400) [20,22], fungi (30–200 and 200–1000 nm) [31], Gram-positive bacteria (20–400 nm), and Gram-negative bacteria (50–250 nm) [4]. However, consistency in particle size range should not be interpreted as definitive evidence that the isolated population consists predominantly of authentic EVs.

To provide an additional bulk compositional descriptor of the isolated material, the protein concentration of each EV-enriched fraction was also measured. Protein concentrations ranged from 72.0 to 339.0 μg/mL, with a mean of 172.1 ± 72.0 μg/mL. The corresponding particle-to-protein ratios ranged from 8.1 × 10^8^ to 6.8 × 10^9^ particles/μg, with an average of 2.47 ± 1.52 × 10^9^ particles/μg (Table 1). These data indicate substantial variation among samples in the relationship between vesicle-sized particle abundance and total co-isolated protein content, further supporting the physicochemical heterogeneity of the EV-enriched fractions.

It should be noted that the material isolated in this study represents an EV-enriched fraction derived from a complex fermentation matrix rather than a rigorously purified population of EVs. As a result, the available data are insufficient to definitively establish EV identity, purity, or origin. Although TEM revealed vesicle-like morphology, such imaging alone cannot exclude the presence of other co-isolated nanoscale materials. Likewise, NTA confirmed the abundance of vesicle-sized particles, but these measurements should not be interpreted as an exact quantification of authentic EVs. In addition, although protein concentrations were measured, the present study did not include systematic quantification of lipids, polysaccharides, or other matrix-derived co-isolated components. Therefore, the current characterization should be regarded as a partial physicochemical description of the isolated material rather than a complete compositional analysis. Furthermore, because red yeast rice contains a diverse microbial community and there are currently no universally recognized EV markers for such heterogeneous microbial systems, this characterization does not definitively specify the origin of the vesicles.

### 3.2. Proteomic Profile of Microbiota-Derived EV-Enriched Fractions from Red Yeast Rice

**Figure 2:**
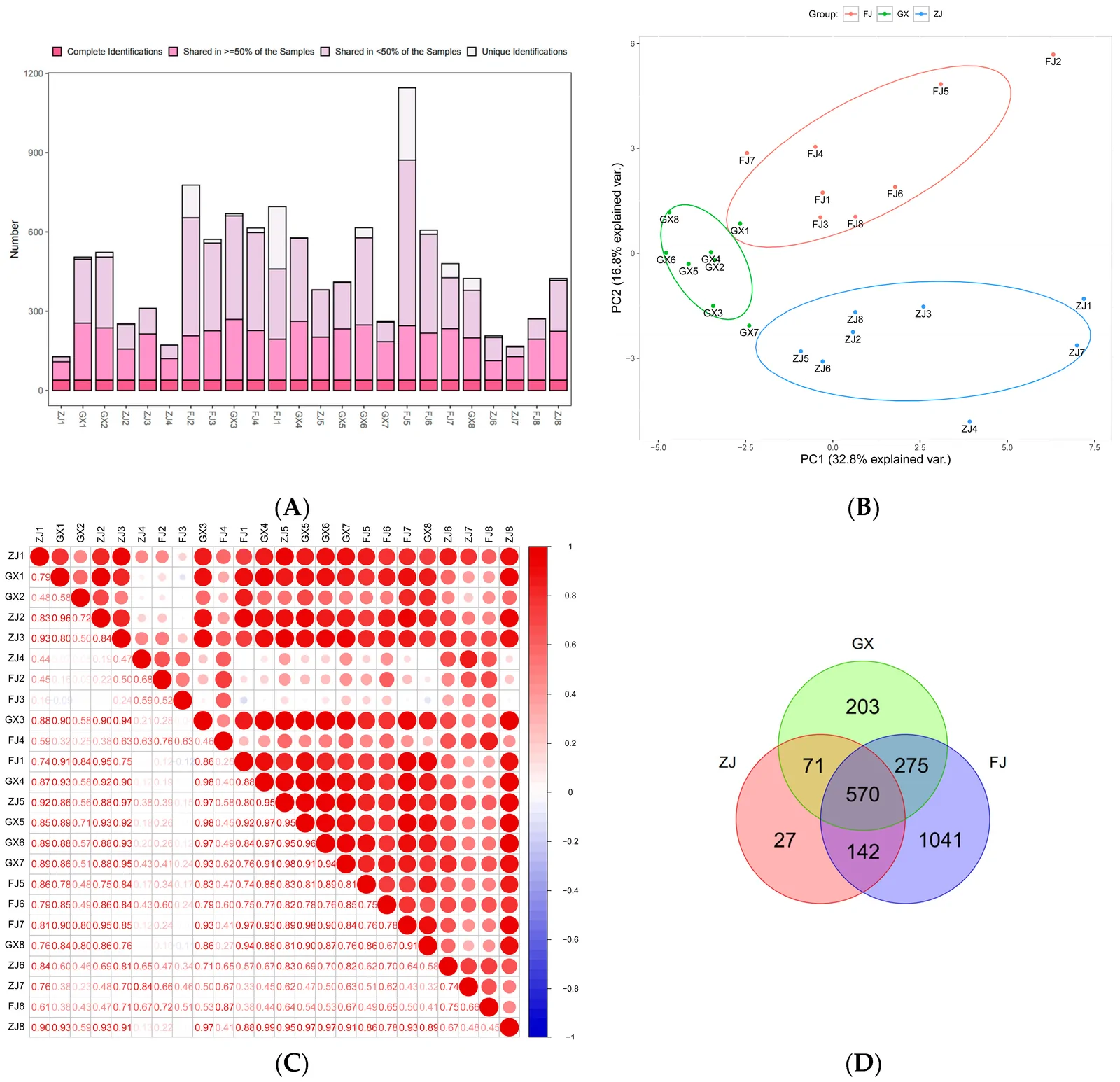
Comprehensive proteomic analysis of microbiota-derived EV-enriched fractions from red yeast rice. (**A**) REV protein identification across three regions. (**B**) Principal component analysis (PCA) of the REV proteins across three regions. (**C**) Correlation heatmap of the REV proteins across three regions. (**D**) Venn diagram depicting the shared and unique proteins identified among the REVs across the three regions.

LC-MS/MS analyses were conducted to determine the protein composition of the REVs. Proteomic identification revealed the presence of 2376 proteins (Figure 2A, Table S2). The most abundant proteins in REVs were major outer membrane lipoprotein (lpp), flagellin (fliC), ubiquitin-40S ribosomal protein S31 (RPS31), outer membrane porin C (ompC), phosphate-binding protein PstS (pstS), chaperonin GroEL 1 (groEL1), sn-glycerol-3-phosphate-binding periplasmic protein UgpB (ugpB), and S-adenosylmethionine synthase (metK). The number of identified and quantified proteins varied among REVs (Table S2). Principal component analysis (PCA) revealed clear separation of samples from different regions, which was further supported by the correlation heatmap analysis (Figure 2B,C). We assessed the effect of the geographic region on REVs proteins (Figure 2D) and identified 2028, 1119, and 810 proteins in the FJ, GX and ZJ groups, respectively, for a total of 2376 proteins. The REVs from FJ contained 1041 unique proteins, whereas only 203 and 27 proteins were unique to the REVs from GX and ZJ, respectively.

Proteomic annotation revealed that the identified proteins were derived from multiple microbial sources, including both bacterial and fungal taxa. These findings align with the complex microbial composition of red yeast rice and suggest that the EV-enriched fraction may contain vesicle-associated or co-isolated components originating from diverse microorganisms. The notably high abundance of Lpp (putatively assigned to bacterial protein; Table S3) and OmpC (putatively assigned to bacterial protein; Table S3) may reflect the contribution of bacterial envelope-associated proteins to the molecular profile of the REV-enriched fraction. OmpC is a well-known trimeric porin that is abundantly secreted within outer membrane vesicles derived from Gram-negative bacteria [32,33]. In addition to Lpp, which plays a crucial role in anchoring the outer membrane and transmitting stress signals [34,35], these structural proteins may help maintain the physical integrity of REVs against environmental turgor pressure during fermentation processes. However, in the present study, the detection of these proteins should not be taken as direct evidence that they structurally define REVs or that they actively maintain vesicle integrity during fermentation. Similarly, the detection of proteins associated with environmental interaction or nutrient acquisition, may indicate that proteins with such annotated functions are present in the EV-enriched fraction. For example, PstS (putatively assigned to bacterial protein; Table S3) and UgpB (putatively assigned to bacterial protein; Table S3) facilitate the efficient uptake of periplasmic phosphate and sn-glycerol-3-phosphate, respectively [36,37,38]. UgpB has also been reported to display chaperone-like activity under specific conditions [39]. Together with the detection of GroEL (putatively assigned to bacterial protein; Table S3), which forms a robust chaperone network (GroEL/UgpB); this observation may raise the possibility that some proteins present in the REV-enriched fraction are functionally related to protein quality control, protecting complex enzymes from misfolding under extracellular stress conditions [40,41]. Nevertheless, the present data do not demonstrate that these proteins remain active within vesicles, form a functional chaperone network, or confer protein-refolding capacity to the REV-enriched fraction. Additionally, the enrichment of flagellin (FliC, putatively assigned to bacterial protein; Table S3), which is canonically associated with bacterial motility, may also serve as a surface adhesin on vesicles, and may hypothetically contribute to vesicle–matrix or vesicle–microbe interactions, but this remains to be experimentally tested [42]. In addition, several identified proteins are involved in basal metabolism and translation. For example, RPS31 (putatively assigned to fungal protein; Table S3) is known to participate in 40S ribosomal subunit assembly and mRNA decoding [43,44]. Its detection in the REV-enriched fraction may indicate the presence of translation-related components or co-isolated intracellular material. Moreover, the abundance of MetK (putatively assigned to bacterial proteins; Table S3), which catalyzes the synthesis of S-adenosylmethionine (SAM), may reflect the metabolic characteristics of the source microorganisms and is broadly consistent with the GO enrichment results related to metabolic processes [45,46]. Taken together, these proteomic features provide a useful basis for generating hypotheses regarding the possible biological roles of red yeast rice-derived EV-enriched fractions, but such roles remain to be experimentally validated.

**Figure 3:**
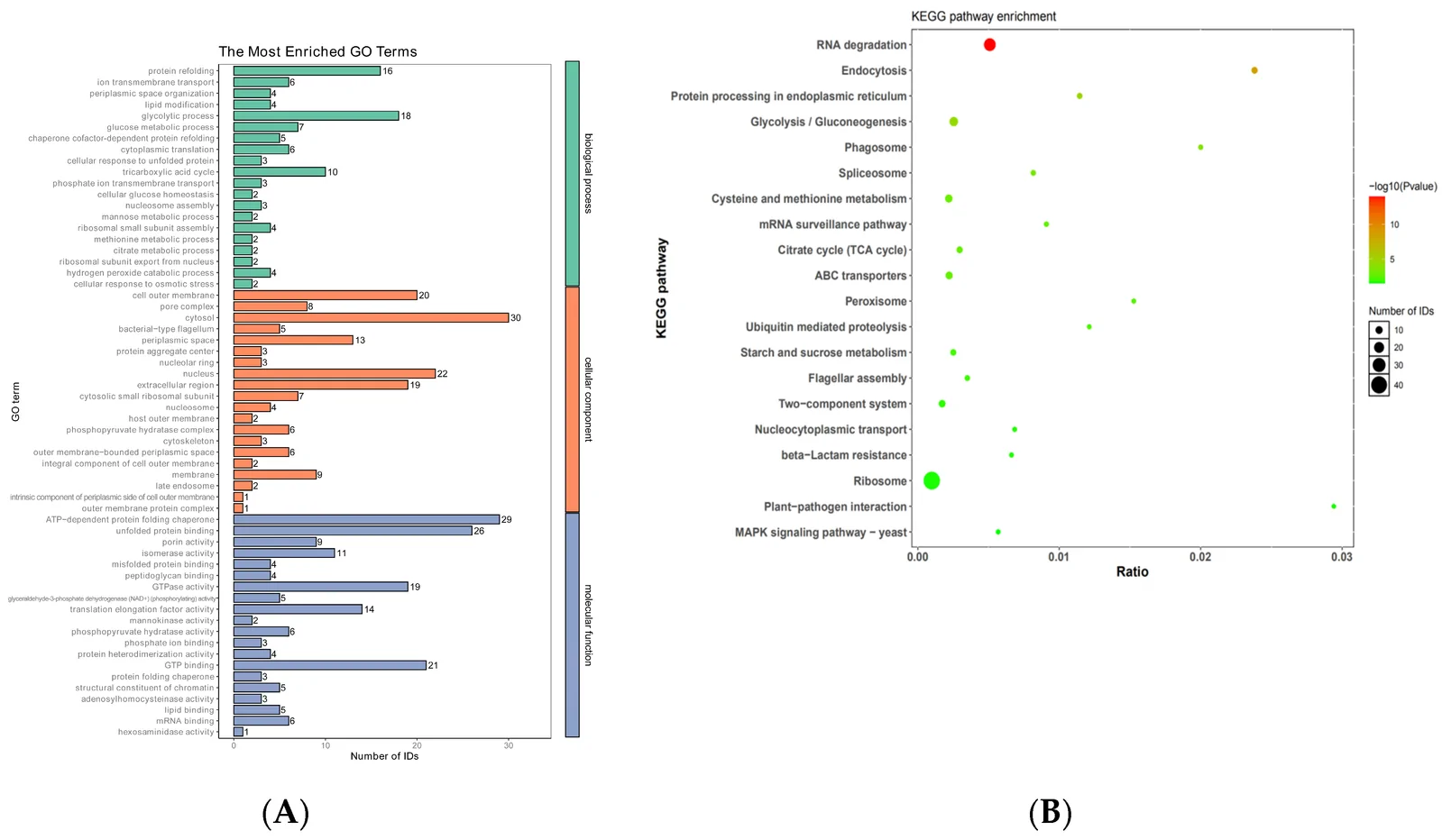

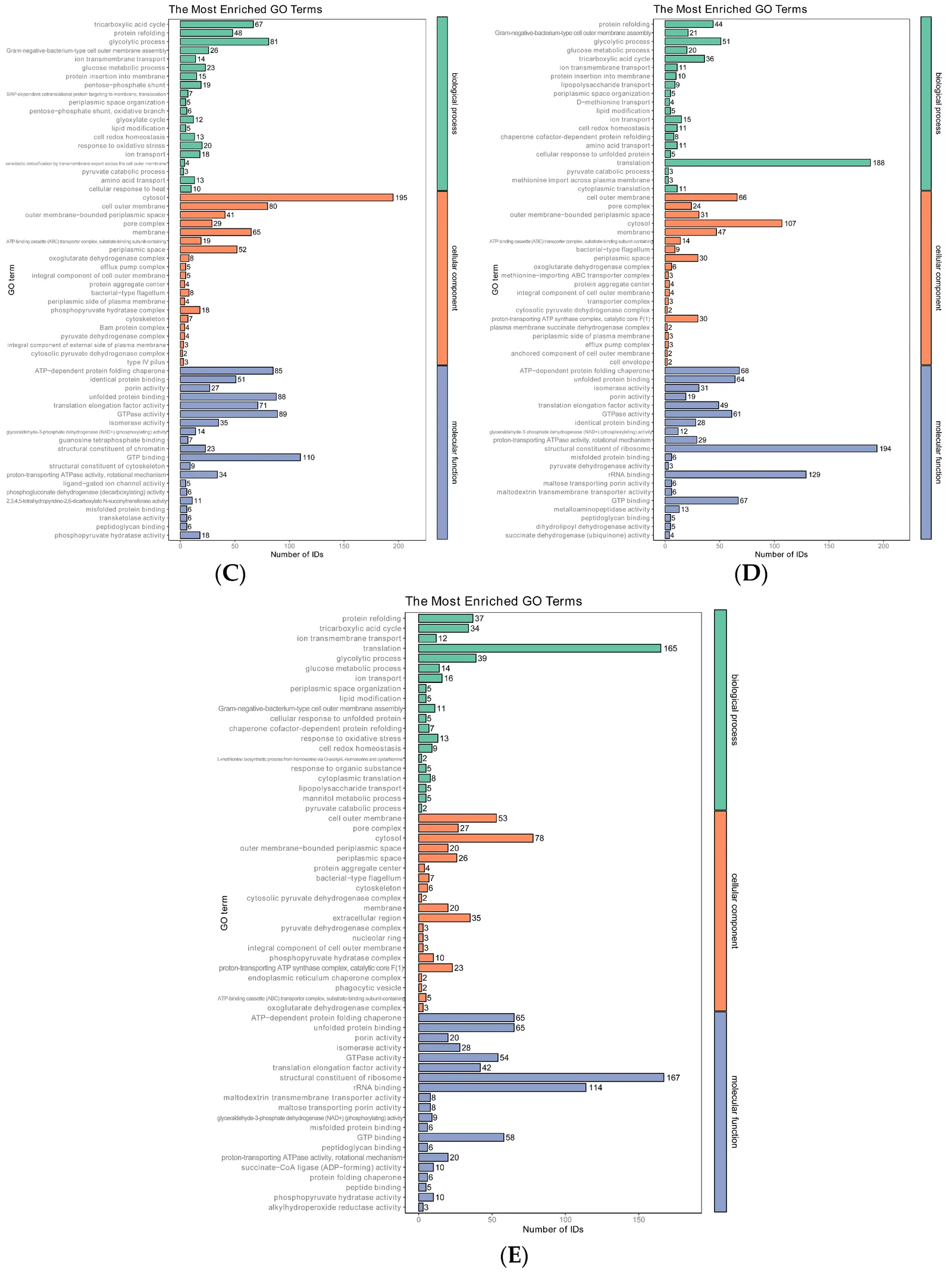
Functional analysis of microbiota-derived EV-enriched fractions from red yeast rice. (**A**) Gene Ontology (GO) functional annotation. (**B**) KEGG pathway enrichment analysis. Functional analysis of microbiota-derived EV-enriched fractions from red yeast rice originating from (**C**) FJ, (**D**) GX, and (**E**) ZJ. Enrichment was performed using a hypergeometric over-representation test. The significance values shown in the plots, including the color scale, represent nominal *p* values.

GO annotations for biological processes were available for 11.5% of the proteins from the REVs. The top 20 enriched GO terms associated with each category are shown in Figure 3. Specifically, a total of 273 annotated proteins were assigned to 287 subcategories of BPs (Table S4), in which “glycolytic process” and “protein refolding” were the two predominant subcategories (Figure 3A). These proteins were further classified into 109 subcategories of the CCs (Table S5), and the largest subcategories were “cytosol” and “nucleus” (Figure 3A). The annotated proteins were mapped into 168 GO terms of the MF category (Table S6), and the two most abundant subcategories were “ATP-dependent protein folding chaperone” and “unfolded protein binding” (Figure 3A). Furthermore, 273 annotated proteins of red yeast rice EVs were analyzed to explore the metabolic pathways associated with these proteins through a KEGG enrichment analysis. The top enriched KEGG pathways were “ribosome” (42), “RNA degradation” (26), “glycolysis/gluconeogenesis” (15), “cysteine and methionine metabolism” (11), and “ABC transporters” (10) (Figure 3B, Table S7).

A total of 570 proteins were shared among the REV-enriched fractions of the three groups, indicating a broadly conserved proteomic profile across red yeast rice samples from different regions. Proteins identified in the REV-enriched fractions were associated with similar annotated biological processes, including ribosomes, RNA degradation, glycolysis/gluconeogenesis, two-component systems, ABC transporters, and cysteine and methionine metabolism (Figure 3C–E). The shared proteome is enriched with highly conserved metabolic and stress-response proteins, such as enolase (eno), glyceraldehyde-3-phosphate dehydrogenase (gapA and gapB), heat shock proteins (ssa2, hsp104, hsp90, and hsp70), and the chaperonins groEL/dnaK. These findings suggest that proteins related to core metabolic and stress-response processes are recurrently detected in REV-enriched fractions from different regions. This pattern may serve as a basis for the hypothesis that the entire microbial consortium utilizes EV-enriched fractions to manage fermentation-induced stress (e.g., heat and oxidative pressure) while sustaining high metabolic flux, a possibility that will require targeted functional studies for validation [47].

### 3.3. Variations in the Proteomic Profiles of Microbiota-Derived EV-Enriched Fractions from Red Yeast Rice Across Different Regions

**Figure 4:**
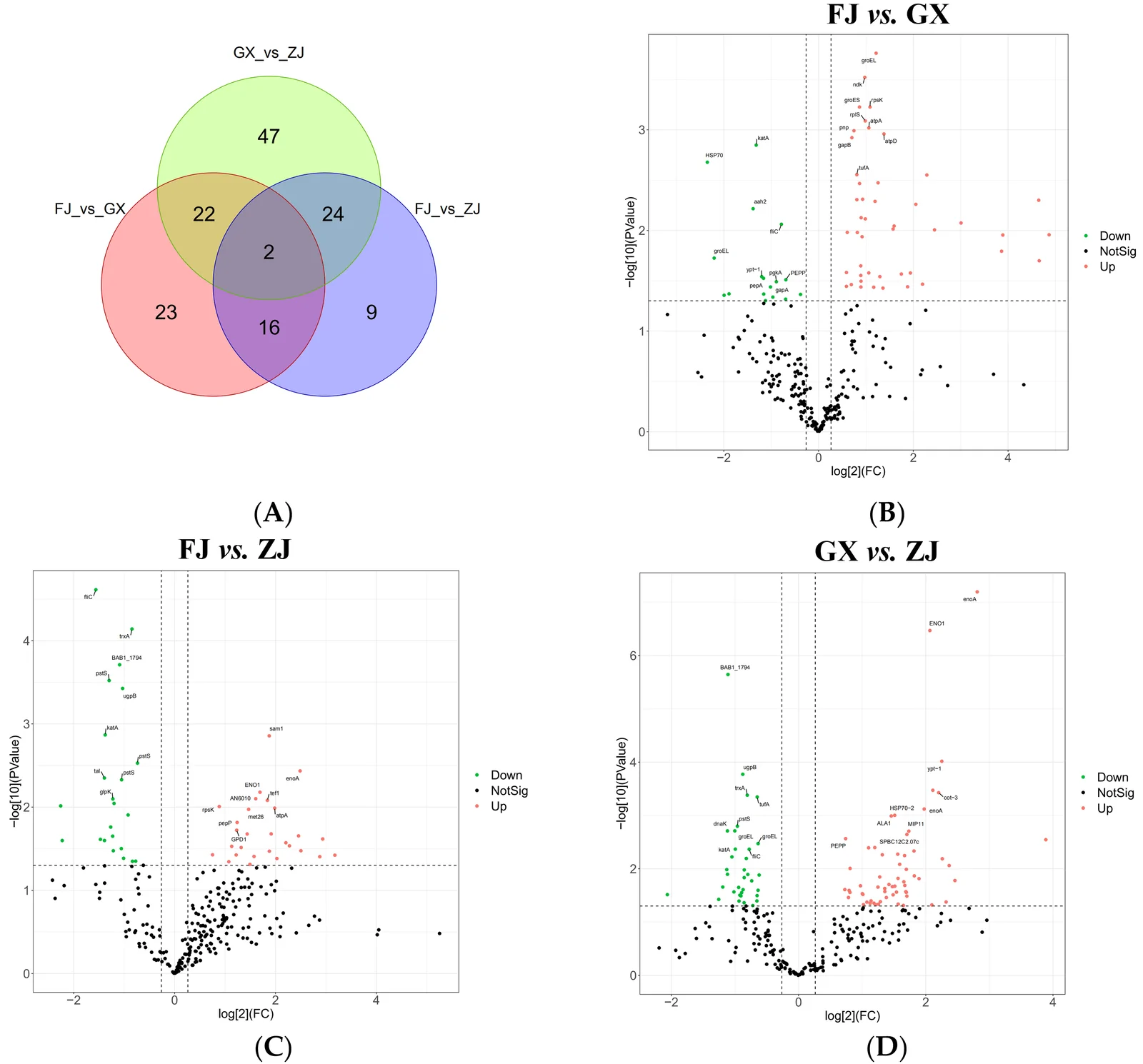
Differential proteomic analysis of the microbiota-derived EV-enriched fractions from red yeast rice across different regions. (**A**) Venn diagram of REV proteins in various regions. Differential abundance volcano plot between (**B**) FJ and GX, (**C**) GX and ZJ, and (**D**) FJ and ZJ. Candidate DEPs were defined using an exploratory screening criterion of nominal *p* < 0.05 and fold change > 1.2 or <1/1.2. Benjamini–Hochberg-adjusted FDR values were additionally calculated for all quantified proteins and are reported in Supplementary Table S8.

An analysis of the differentially expressed proteins (DEPs) in REVs from various regions was conducted. Using the exploratory screening criteria of nominal *p* < 0.05 and FC > 1.2 or <1/1.2, a total of 143 candidate DEPs were identified between any two of the three regions (Table S8). The results of the differential expression analysis (Figure 4A) revealed 95 candidate DEPs between GX and ZJ, representing approximately 4% of the REV proteins. Additionally, 63 candidate DEPs were identified between the FJ and GX groups, accounting for approximately 2.7% of the REV proteins, whereas 51 candidate DEPs were detected between the FJ and ZJ groups, representing approximately 2.1% of the REV proteins. After Benjamini–Hochberg correction for multiple testing, 24, 10, and 5 proteins remained significant at FDR < 0.05 in the GX vs. ZJ, FJ vs. GX, and FJ vs. ZJ comparisons, respectively (Table S8). These findings suggest that only a limited subset of REV proteins showed statistically robust regional differences after multiple-testing correction. Therefore, the regional proteomic differences and associated enrichment patterns reported here should be considered hypothesis-generating and interpreted with caution.

As shown in Table S8 and Figure 4B, 63 candidate DEPs were identified between the FJ and GX groups. Compared with those in the GX group, 46 proteins in the FJ group were upregulated. These proteins include groEL, ndk, groES, rpsK, rplS, pnp, gapB, atpA, atpD, and tufA. Moreover, 17 proteins in the FJ group were downregulated compared with those in the GX group. These proteins include HSP70, katA, aah2, fliC, ypt-1, pgkA, PEPP, pepA, and gapA. Fifty-one candidate DEPs were identified between the FJ and ZJ groups (Figure 4C). Twenty-three proteins were upregulated in the ZJ group compared with those in the FJ group. These proteins include fliC, trxA, BAB1_1794, pstS, ugpB, kata, tal, and glpK. In contrast, 28 proteins were downregulated in the ZJ group compared with those in the FJ group. These proteins include sam1, enoA, ENO1, AN6010, tef1, met26, pepP, rpsk, GPD1, and atpA. A total of 95 candidate DEPs were identified between the GX and ZJ groups (Figure 4D). Compared with those in the ZJ group, 61 proteins in the GX group were upregulated. These proteins include enoA, ENO1, ypt-1, cot-3, HSP70-2, enoA, ALA1, MIP11, SPBC12C2.07c, and PEPP. In contrast, 34 proteins in the GX group were downregulated compared with those in the ZJ group. These proteins include BAB1_1794, ugpB, trxA, tufa, dnaK, pstS, kata, groEL, and fliC.

Carbon and nitrogen metabolism are central to cellular energy production and biosynthesis. In the ZJ group, proteins such as glycerol kinase (glpK) and transaldolase (tal) showed higher abundance. Given their known roles in glycerol metabolism and the nonoxidative pentose phosphate pathway, these differences may reflect variation in carbon metabolic potential among groups, although protein abundance alone does not demonstrate pathway activity or metabolic flux. Glycerol is an important metabolite and osmolyte that can serve as a precursor for phospholipid biosynthesis and is often associated with cellular responses to environmental change [48,49]. Transaldolase (tal) is a ubiquitous enzyme in central carbon metabolism that catalyzes the transfer of a dihydroxyacetone (DHA) moiety from a ketose donor to an acceptor aldehyde in the nonoxidative pentose phosphate pathway [50]. Conversely, proteins related to amino acid metabolism and specific carbon metabolism (e.g., sam1, ENO1, met26, and GPD1) showed lower abundance in the ZJ group. S-Adenosylmethionine synthetase (sam1) and 5-methyltetrahydropteroyltriglutamate-homocysteine methyltransferase (met) are essential for methylation reactions, methionine metabolism [51,52], and GABA biosynthesis [53]. In addition, BAB1_1794 was more abundant in the ZJ group. As a homolog of Leu/Ile/Val-binding proteins, BAB1_1794 has been annotated as a periplasmic binding protein involved in branched-chain amino acid transport, suggesting possible differences in transport-related protein representation among groups, although transport activity itself was not measured. Furthermore, specific hydrolytic enzymes such as Aah2 and PepA showed lower abundance in the FJ group. Aah2 (Alpha-amylase 2) catalyzes the endohydrolysis of (1→4)-alpha-D-glucosidic linkages in polysaccharides containing three or more such units. Penicillopepsin-2 (pepA) is an aspartic endopeptidase with broad specificity for protein hydrolysis [54]. These abundance differences indicate compositional variation in REV-associated enzymes among regions, but they do not by themselves establish distinct nutrient utilization strategies or exudation profiles.

Notably, differences were also observed in the abundance of highly conserved translational and structural proteins. Specifically, the FJ group presented higher levels of 30S ribosomal protein S11 (rpsK) and ATP synthase subunit (atpD). Because these proteins are associated with translation and energy conversion, their differential representation may indicate variation in the REV proteomic signatures linked to these core cellular processes; however, it should not be taken as direct evidence of increased protein translation or energy transduction. Ribosomal proteins, which are located in the large and small ribosomal subunits (such as rpsK and 30S ribosomal protein S11), are likely to be conserved across species; however, they may also exhibit sufficient sequence variability to trace evolutionary relationships among different organisms [55]. ATP synthase, a multisubunit membrane-associated protein complex found in bacterial, mitochondrial, and chloroplast membranes, plays a crucial role in energy conversion by synthesizing the majority of cellular ATP from ADP and inorganic phosphate [56,57].

Interestingly, many of the candidate DEPs identified, particularly those related to translation and glycolysis (e.g., tufA, tef1, GPD1, and ENO1), are well-known “moonlighting” proteins. While they play canonical roles in intracellular protein synthesis or carbon metabolism, they are frequently packaged into extracellular vesicles in bacteria and yeasts, where they perform secondary functions such as cell signaling, cytoskeletal remodeling, and the modulation of adherence or virulence [15,18]. EF-Tu (elongation factor Tu, tufA), a protein best known for its role in protein synthesis, is a very old and conserved protein. All bacteria possess an EF-Tu-related cytoskeleton [58]. Studies have shown that EF-Tu is associated with the cell surface and performs “moonlighting” functions in a wide range of microbes, including roles in type VI secretion and contact-dependent growth inhibition [59]. Translation elongation factor-1 alpha (EF-1α, tef1), the eukaryotic (and archaebacterial) counterpart to elongation factor Tu (EF-Tu) in bacteria, interacts with aminoacyl-tRNAs (aa-tRNAs) in a guanosine triphosphate (GTP)-binding-dependent manner and delivers them to the acceptor site of the 80S ribosome. EF-1α is known to be involved in diverse cellular processes through moonlighting functions, such as cytoskeletal remodeling, protein folding and degradation, proteolysis, cell signaling modulation, cell growth control, nuclear export processes, apoptosis, and the cell cycle [60]. Therefore, the between-group variation observed for these moonlighting proteins may be relevant to extracellular or host-/environment-facing functions of REVs. However, the present data do not directly demonstrate that these vesicles regulate external biotic interactions or environmental remodeling.

The REV proteomes also differed in the representation of proteins related to proteostasis and redox balance. In the GX group, Ypt1 and HSP70-2 were more abundant. These proteins are known to participate in protein folding, trafficking, and chaperone-related functions, suggesting variation in the proteostasis-related protein content of REVs among groups, although no direct measurements of chaperone activity or stress tolerance were performed. Ypt1p (GTP-binding protein Ypt1) is part of the Ypt/Rab GTPase family and is involved primarily in regulating intracellular trafficking pathways [61]. Specifically, YPT1 facilitates protein transport from the endoplasmic reticulum (ER) to the Golgi apparatus. Ypt1p can reversibly function as a GTPase at relatively low temperatures and, owing to structural changes, can act as a molecular chaperone at relatively high temperatures. This chaperone activity significantly contributes to increasing yeast cell tolerance to heat stress [62]. Hsp70 chaperones are highly conserved across all organisms and are essential for maintaining proteostasis. Bacterial dnaK is a member of the Hsp70 family and plays a crucial role in assisting in protein folding and stability [63,64]. On the other hand, redox-related proteins also varied among groups. Thioredoxin 1 (trxA), which was more abundant in the ZJ group, is a conserved redox-regulatory protein that contributes to maintaining cellular redox homeostasis by reducing oxidized proteins [65]. Trx has dual functions as both an antioxidant and a cofactor for various enzymes and transcription factors [66]. Moreover, catalase (katA), a primary H_2_O_2_ detoxification enzyme that is often associated with oxidative stress responses [67,68] and has previously detected in bacterial EVs [69], showed lower abundance in the FJ group. This pattern was accompanied by differences in the abundance of iron–sulfur cluster-related proteins, including IscS and AN6010 [70]. As cofactors, iscS binds to proteins and enzymes that are essential for numerous biological processes, including DNA synthesis and repair, mitochondrial function, and gene transcription and translation [71]. Together, these observations indicate differences in the abundance of REV-associated proteins linked to redox regulation and proteostasis. However, they should not be interpreted as direct evidence that the source organisms were experiencing different levels of oxidative stress.

EVs encapsulate signature surface components from their cells of origin, often inheriting and extending the biological functions of the parent cell. This specificity underscores the potential of EVs as biomarkers in various contexts [72]. Notably, our findings indicate that the FJ samples exhibit elevated levels of rpsK and atpD; the GX samples contain higher levels of ypt-1, HSP70-2, ALA1, and MIP11; and the ZJ samples contain higher levels of trxA, BAB1_1794, pstS, ugpB, tal, and glpK. *Saccharomyces cerevisiae* proteomic data available at PaxDb (www.pax-db.org) indicated that GPD1 is among the most abundant proteins in yeast cells, suggesting that it may not serve as a suitable candidate region-associated protein. Conversely, HSP70 has been identified as a potential marker for fungal EVs [73]. Additionally, given its presence in exosomes derived from mammalian cells, HSP70 may function as a general marker for eukaryotic EVs [74]. However, owing to its high abundance and presence in multiple cellular compartments, HSP70 alone is not an ideal specific marker for fungal EVs [75]. Therefore, rpsK and atpD are good candidates for use as candidate region-associated proteins for the FJ region; ypt-1, ALA1, and MIP11 are good candidates for use as candidate region-associated proteins for the GX region; and trxA, BAB1_1794, pstS, ugpB, tal, and glpK are good candidates for use as candidate region-associated proteins for the ZJ region.

### 3.4. Variations in Biological Functions Determined Using Gene Ontology Analysis and KEGG Pathway Enrichment Analysis of Proteins in Microbiota-Derived EV-Enriched Fractions from Red Yeast Rice Obtained from Different Regions

**Figure 5:**
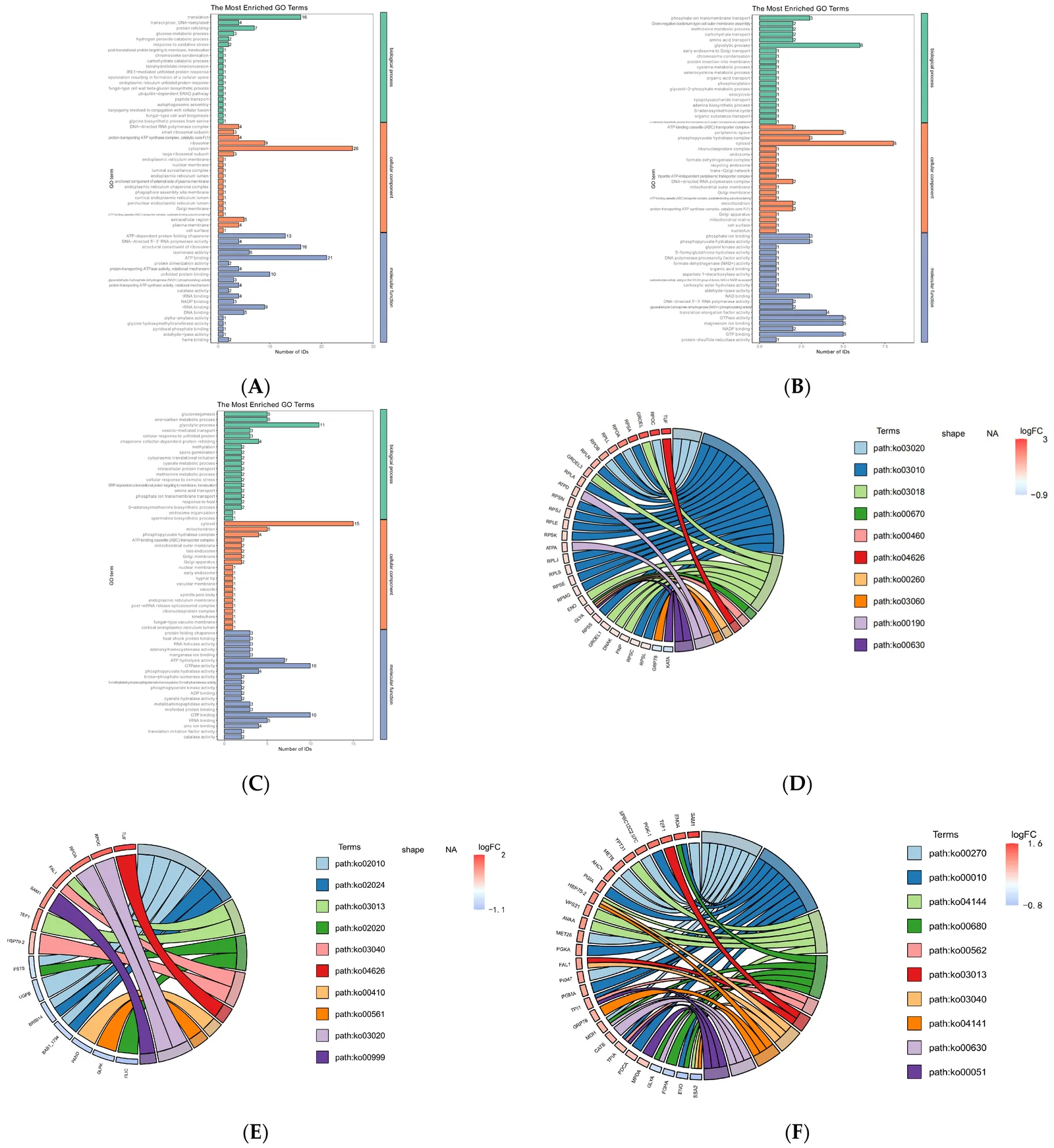
Functional analysis of the REV proteome across different regions. GO functional annotation analysis of candidate differentially expressed proteins between (**A**) FJ and GX, (**B**) GX and ZJ, and (**C**) FJ and ZJ. KEGG pathway analysis of candidate DEPs between (**D**) FJ and GX, (**E**) GX and ZJ, and (**F**) FJ and ZJ. Candidate DEPs were defined using nominal *p* < 0.05 and fold change > 1.2 or <1/1.2 as an exploratory screening criterion. Enrichment was performed using a hypergeometric over-representation test. The significance values displayed in the enrichment plots represent nominal *p* values. In the chord diagrams, candidate DEPs are displayed on the left and KEGG pathway terms on the right. Protein colors indicate logFC values, with red representing upregulation and blue representing downregulation. Links between proteins and pathways indicate protein enrichment in the corresponding KEGG pathways.

We conducted Gene Ontology (GO) annotation and KEGG pathway enrichment analyses of the 143 candidate DEPs identified across the three regions (Figure 5A–C). With respect to the BP annotations, candidate DEPs identified in the FJ vs. GX comparison were significantly associated with terms related to translation, protein refolding, transcription, DNA-templated, and glucose metabolic processes. Moreover, in the FJ vs. ZJ comparison, the candidate DEPs were significantly enriched in BP terms including glycolytic processes and phosphate ion transmembrane transport. In addition, in the GX vs. ZJ comparison enriched BP terms included glycolytic process, gluconeogenesis, one-carbon metabolic process, and chaperone cofactor-dependent protein. Overall, metabolism-related and protein biosynthesis-related BP terms, particularly glycolytic process, protein refolding, and translation, were commonly enriched among the three comparisons. With respect to CCs (Figure 5A–C), more candidate DEPs were annotated to the cytoplasm or cytosol pathways. With respect to MFs, candidate DEPs identified in the FJ vs. GX comparison were enriched in terms such as ATP binding, structural constituents of ribosomes, ATP-dependent protein folding chaperones, and unfolded protein binding. In the FJ vs. ZJ comparison, enriched MF terms included GTPase activity, magnesium ion binding, and GTP binding. In the GX vs. ZJ comparison, enriched MF terms included GTPase activity, GTP binding, ATP hydrolysis activity, and tRNA binding.

A total of 98 candidate DEPs were enriched in 67 KEGG pathways, including biosynthesis of ribosomes, RNA degradation, ABC transporters, and cysteine and methionine metabolism. As shown in Figure 5D and Figure S1A, KEGG enrichment analysis indicated that the candidate DEPs identified in the FJ vs. GX comparison were significantly overrepresented in pathways annotated as ribosomes (path:ko03010, which includes proteins such as rplS, rpsL, rpsS, rplN, rplL, rpmG, rpsJ, rpsE, rpsN, rplA, rplL, and rpsA), RNA degradation (path:ko03018, which includes proteins such as dnaK, groEL, groEL, dnaK, dnaK, groEL, pnp, groEL1, eno, and groEL3), RNA polymerases (path:ko03020, which includes proteins such as rpoA, rpoC, and rpoB), oxidative phosphorylation (path:ko00190, which includes proteins such as atpA, atpA, atpA, and atpD), and glyoxylate and dicarboxylate metabolism (path:ko00630, which includes proteins such as glyA and katA). As shown in Figure 5E and Figure S1B, KEGG enrichment analysis indicated that the candidate DEPs identified in the FJ vs. ZJ comparison were significantly overrepresented in pathways annotated as ABC transporters (path:ko02010, including proteins such as pstS, pstS, BAB1_1794, BR0014, ugpB, ugpB, and pstS), two-component systems (path:ko02020, including proteins such as pstS, pstS, fliC, and pstS), nucleocytoplasmic transport (path:ko03013, including proteins such as fal1 and tef1), quorum sensing (path:ko02024, including proteins such as BAB1_1794 and BR0014), and spliceosomes (path:ko03040, including proteins such as fal1 and HSP70-2). As shown in Figure 5F and Figure S1C, the candidate DEPs identified in the GX vs. ZJ comparison were significantly enriched in KEGG pathways annotated as glycolysis/gluconeogenesis (path:ko00010, including proteins such as pgkA, pgk-1, pgmA, pdcA, enoA, eno, TPI1, tpiA, and pgiA), cysteine and methionine metabolism (path:ko00270, including proteins such as ahcY, mdh, ahcY, pi047, sam1, MET6, SPBC12C2.07c, and met26), endocytosis (path:ko04144, including proteins such as avaA, ssa2, VPS21, YPT31, and HSP70-2), methane metabolism (path:ko00680, including proteins such as fghA, mdh, glyA, enoA, and eno), and inositol phosphate metabolism (path:ko00562, including proteins such as TPI1 and tpiA).

### 3.5. Metaproteomic Analysis Provided Insights into the Major Microbial Taxonomic Classifications and Functions of Proteins in Microbiota-Derived EV-Enriched Fractions from Red Yeast Rice

A total of 273 high-confidence proteins matching two or more unique peptides were identified in all the samples. Among the 273 proteins, 62.3% were classified as belonging to bacteria, 37.4% as belonging to eukaryotes, and 0.4% as belonging to archaea (Table S3). Bacteria contributed the highest relative REV protein abundance in the three regions, and the relative abundances of bacterial REV proteins in FJ, GX, and ZJ were 66.5%, 63.6%, and 57.6%, respectively. The relative abundances of eukaryotic REV proteins in FJ, GX, and ZJ were 29.7%, 30.6%, and 36.4%, respectively. The dominant prokaryotic and eukaryotic groups and their relative REV protein abundances in the three regions are summarized in Table S9.

**Figure 6:**
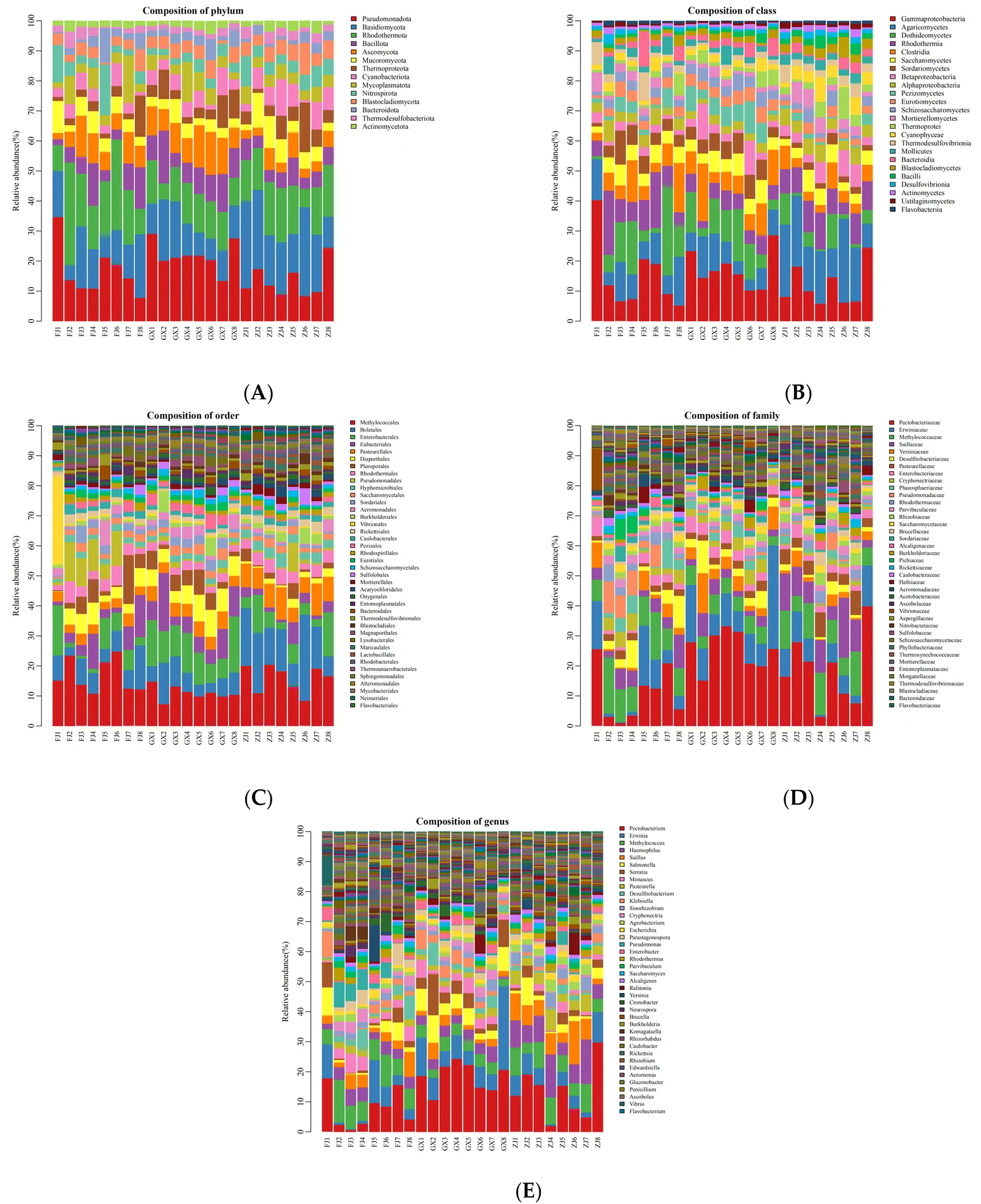
Metaproteomic analysis of microbial community structures in the microbiota-derived EV-enriched fractions from red yeast rice. Taxonomic profiles at the (**A**) phylum, (**B**) class, (**C**) order, (**D**) family, and (**E**) genus levels.

In the taxonomic analysis, taxonomic summarization was based on the representative/master protein reported in the Protein ID of the FragPipe/Philosopher output, and indistinguishable proteins were not counted separately. A total of 14 phyla and 87 genera were identified within the REV metaproteome across all datasets. The most predominant taxa are presented in Figure 6 (rank data) and Supplementary Tables S10–S13 (relative abundance data). At the phylum level, *Pseudomonadota* was the most abundant phylum, with an average relative abundance of approximately 17.3%. The second- and third-most abundant phyla were *Basidiomycota* and *Rhodothermota*, each with an average relative abundance of approximately 15%. *Ascomycota* and *Bacillota* were ranked fourth and fifth, with relative abundances of approximately 7.4%. At the genus level, *Pectobacterium* was identified as the genus with the highest mean relative abundance, followed by *Erwinia* and *Methylococcus*. In addition, several other genera, including *Haemophilus*, *Suillus*, *Salmonella*, *Serratia*, *Monascus*, *Pasteurella*, *Desulfitobacterium*, *Klebsiella*, *Sinorhizobium*, *Cryphonectria*, *Agrobacterium*, *Escherichia*, *Parastagonospora*, *Pseudomonas*, and *Enterobacter*, were relatively abundant, each with a mean relative abundance exceeding 1.5%.

**Figure 7:**
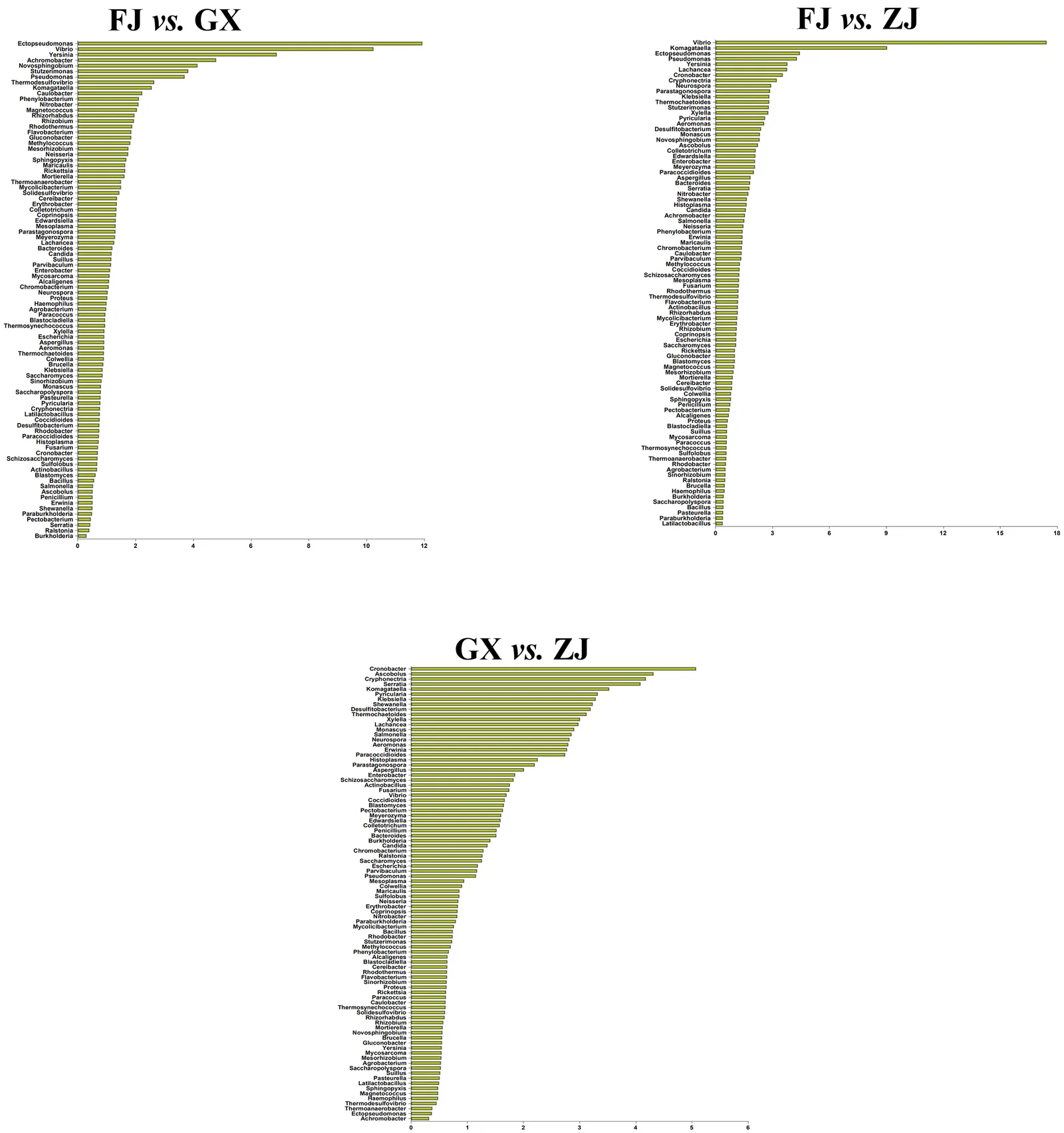
Analysis of predominant genera within the REV proteome across different regions.

We subsequently performed a comparative analysis of the metaproteomic profiles across the three regions to explore region-associated taxonomic pattern on the basis of the mean protein quantification values (Table S14). For each annotated genus, the protein quantification values of all associated proteins were summed and averaged within each regional group. Genera were considered region-associated when they showed relatively higher rank and higher mean protein abundance than in the other two regional groups. As shown in Figure 7, protein annotations associated with *Ectopseudomonas*, *Yersinia*, *Vibrio*, *Pseudomonas*, and *Komagataella* were more prominent in the FJ group. Moreover, annotations associated with *Pectobacterium*, *Erwinia*, *Serratia*, *Salmonella*, and *Ascobolus* were relatively more abundant in the GX group. In addition, annotations associated with *Haemophilus*, *Pasteurella*, *Brucella*, *Latilactobacillus*, and *Saccharopolyspora* were more prominent in the ZJ group.

Another primary aim of this study was to examine taxon-associated functional annotations represented in the metaproteomic profiles of red yeast rice. Therefore, peptide abundance data were aggregated based on taxonomic and functional annotations, resulting in lists of phylum-specific functional features (including KEGG KO functions and pathways), along with their relative abundances and rankings (Table S15). As illustrated in Figure S2A,B, most of the top-ranked phylum-associated functions were linked to *Pseudomonadota*, including several ribosomal proteins and outer membrane proteins annotated to ABC transporters, oxidative phosphorylation, and two-component systems. Notably, *Ascomycota* was the second-highest ranked phylum and included several proteins annotated to glycolysis/gluconeogenesis and cysteine and methionine metabolism.

During the early stages of fermentation, the degradation of raw material matrices is crucial. Our findings indicate that proteins assigned to *Ascomycota* were mainly annotated to functions related to carbohydrate transport and metabolism, suggesting a potential contribution of fungal members to substrate transformation. Furthermore, protein annotations associated with genera such as *Pectobacterium* and *Erwinia* may be relevant to plant cell wall degradation, which is consistent with previous reports that these genera possess a vast array of plant cell wall-degrading enzymes (e.g., pectate-degrading enzymes), which cleave pectin into small oligogalacturonates, often packaging these enzymes into extracellular vesicles [76,77,78]. Additionally, previous studies have reported that *Monascus* and *Klebsiella* act as major contributors of enzymes related to starch hydrolysis [79], whereas *Sinorhizobium* can produce carboxymethyl cellulase (CMCase) to degrade plant cell structures and supply essential nitrogen through nitrogen fixation; this process also provides the necessary energy to support the metabolic activities of other beneficial bacteria, such as *Acetobacter* [80,81]. Although these literature-based functions are consistent with possible roles in substrate breakdown and nutrient supply, our metaproteomic data do not directly demonstrate that these taxa performed these activities in the analyzed samples. Therefore, these interpretations should be considered putative and hypothesis-generating.

In addition to substrate degradation, several taxa detected in the metaproteomic dataset may be associated with biochemical transformations relevant to the quality of red yeast rice. *Monascus* spp., the primary starter fungi, have been widely reported to tolerate ethanol and lactic acid and maintain stable abundance throughout the brewing process [82]. *Monascus* can metabolize and produce a variety of physiologically active secondary compounds, including monacolin K, pigments, γ-aminobutyric acid, and lovastatin, which may offer health benefits [83]. Similarly, proteins annotated to bacterial genera such as *Pseudomonas*, *Klebsiella*, and *Serratia* were also detected, suggesting their potential association with metabolic processes relevant to flavor formation. Metaproteomic analysis indicated that several proteins annotated to *Pseudomonas* were mapped to functions related to alcohol/aldehyde interconversion, fatty acid synthesis and degradation, and amino acid metabolism, including qedA (quinoprotein ethanol dehydrogenase), qgdA (quinohemoprotein alcohol dehydrogenase), exaC (acetaldehyde dehydrogenase), betB (betaine aldehyde dehydrogenase), calB (probable coniferyl aldehyde dehydrogenase), fadB (fatty acid oxidation complex), acpP (acyl carrier protein), fabZ (3-hydroxyacyl-[acyl-carrier-protein] dehydratase), fabG (3-oxoacyl-[acyl-carrier-protein] reductase), and fabI (Enoyl-[acyl-carrier-protein] reductase [NADH]). These annotation-based associations suggest a possible contribution of *Pseudomonas*-related proteins to the generation of volatile precursors or other flavor-related metabolites. This interpretation is broadly consistent with previous studies reporting that *Pseudomonas* can produce volatile organic compounds such as butanol-1, methyl thiolacetate, dimethyl disulfide, 2-heptanone, 1-undecene, 2-nonanone, and 2-undecanone [84], and that its presence may correlate with octanoic acid production [85]. With respect to *Klebsiella*, the detected proteins annotations included tyrB (tyrosine aminotransferase), dhaT (1,3-propanediol dehydrogenase), oadA (oxaloacetate decarboxylase), gcvP (glycine dehydrogenase), fabA (3-hydroxydecanoyl-[ACP] dehydratase), acpP (acyl carrier protein), pgk (phosphoglycerate kinase), and fbp (fructose-1,6-bisphosphatase). These proteins are potentially involved in amino acid metabolism, alcohol-related pathways, central carbon metabolism, and fatty acid precursor synthesis. In particular, aromatic amino acid transformation and fatty acid metabolism are closely linked to the generation of volatile precursors in fermented systems. Previous studies have shown that *Klebsiella* can contribute genes (e.g., amino acid decarboxylase, phenylacetaldehyde dehydrogenase, and alcohol dehydrogenase) involved in the formation of higher alcohols such as isoamyl alcohol and isobutyl alcohol [86,87,88]. In the present study, these annotation-based associations suggest a possible link between *Klebsiella*-related proteins and metabolic processes relevant to flavor precursor formation. Likewise, proteins annotated to *Serratia* were detected in the fermentation system. Representative proteins, including thrA (bifunctional aspartokinase/homoserine dehydrogenase), thrC (threonine synthase), pgk (phosphoglycerate kinase), and gap (glyceraldehyde-3-phosphate dehydrogenase), which are potentially involved in amino acid biosynthesis and carbon metabolism processes. These pathways may influence the production and conversion of metabolites that serve as precursors of volatile compounds. Previous studies have demonstrated that *Serratia* is capable of producing volatile organic compounds, such as isopentanol dimethyl disulfide, 2-heptanone 1,5-dimethylpyrazine, s-methyl thiooctanoate [84], 2,3-butanediol and acetoin [89,90]. In our dataset, the detection of *Serratia*-associated proteins is consistent with a potential contribution to flavor-related metabolism.

Moreover, the metaproteomic dataset suggested the presence of protein annotations linked to taxa previously reported to be associated with the biosynthesis of high-value health-promoting metabolites. For example, *Desulfitobacterium* and *Suillus* have been reported to be associated with the biosynthesis of vitamin K2 [91] and antioxidant and anticholesteric properties [92]. Additionally, proteins annotated to *Pasteurella* and *Agrobacterium* may be relevant to the synthesis of functional exopolysaccharides, as previous studies have reported hyaluronan synthase from *Pasteurella* [93] and water-soluble glucans production by *Agrobacterium* [94,95]. Interestingly, some taxa detected in the dataset may also have positive effects on host health upon consumption. For example, nitrate-reducing microorganisms such as *Haemophilus* have been suggested to contribute to nitric oxide precursor formation, which have been linked to improved vascular health and cognitive performance via the gut–brain axis [96,97]. In addition, *Parastagonospora* has been reported to be associated with host energy metabolism [98]. These microecological interactions may suggest that red yeast rice fermentation not only results in the development of rich flavors but also acts as a reservoir for bioactive compounds and potential prebiotic/probiotic targets. Nevertheless, these observations are based primarily on prior literature and taxonomic annotation, and they should not be interpreted as direct evidence that the corresponding microorganisms in red yeast rice fermentation exert these effects after consumption. Rather, they indicate hypotheses that may warrant targeted validation in future studies.

The REV proteome also provides information potentially relevant to microbial interactions and safety risks during fermentation. For instance, previous studies have shown that *Pseudomonas* exhibits antagonistic activity against specific lactic acid bacteria and various yeast species and plays a regulatory role in maintaining the microecological balance [99]. Conversely, the detection of annotations associated with genera such as *Salmonella*, *Escherichia*, and *Enterobacter* raises concerns regarding fermentation safety and off-flavors, because these taxa have previously been reported to be negatively associated with desirable flavor compounds, including phenylethyl alcohol, isobutanol, and ethyl acetate, during Hongqu rice wine brewing [85,100]. These negative associations are more likely to reflect microbial competition, variations in fermentation conditions, or community succession dynamics, whereby the proliferation of desirable fermentative microorganisms is associated with increased flavor compound production and a reduction in the abundance of undesirable or contaminant-associated bacteria. More importantly, these genera have been reported to be positively associated with the accumulation of biogenic amines such as tyramine, putrescine, histamine, and cadaverine [87]. *Desulfitobacterium* has also been identified in previous work as a contributor to the genes encoding tryptophan decarboxylase [101]. Thus, monitoring protein annotations associated with these taxa in RYR may serve as an effective strategy for evaluating and controlling safety and flavor quality during the fermentation process, although targeted validation would be required to confirm their specific roles.

The findings derived from the REV metaproteomic analysis revealed diverse protein annotations associated with both prokaryotic and eukaryotic microorganisms, supporting the view that multiple microbial members may contribute to the extracellular vesicle-associated proteome in red yeast rice fermentation. These findings improve our understanding of the taxonomically associated protein landscape and the potential functional complexity of the fermentation microbiome. Notably, previous studies have shown that some extracellular vesicle-associated proteins can retain their native activity; for instance, glycosidases and proteases from *Lactobacillus*-derived EVs have been shown to maintain their enzymatic function [18]. Additionally, emerging research has indicated that extracellular vesicle-associated proteins may play direct roles in interspecies and cross-kingdom interactions. Huang et al. demonstrated that extracellular vesicles from rice can transfer host defense proteins to the fungal pathogen *Rhizoctonia solani*, resulting in reduced disease progression [6]. While this mechanism has not been confirmed in red yeast rice fermentation, these findings highlight the potential significance of EV-mediated protein transfer as an underexplored pathway that could influence microbial interactions and functional processes within complex fermentation environments. Therefore, REVs and their protein cargoes may influence the fermentation microenvironment and may also affect the gut microenvironment after consumption, warranting further investigation in future studies. These aspects require dedicated functional validation in future studies.

## 4. Conclusions

In summary, this study is the first to report EV-enriched fraction derived from red yeast rice. The size distributions of these EV-enriched fractions are consistent with those observed for extracellular vesicles from other yeasts, molds, and bacteria. Our study revealed the protein composition of the red yeast rice-derived EV-enriched fraction. Proteomic analysis revealed that these vesicle-associated proteins were enriched in membrane-associated components, functions associated with vesicle-mediated transport, carbohydrate metabolism, and their respective substrates. These findings improve our understanding of the protein profiles and properties of EV-enriched fractions obtained from red yeast rice. Furthermore, our results contribute to the existing knowledge base by identifying numerous differentially expressed proteins with significant variations. The bioinformatics analysis also provided insights into the regional dynamics of the REV-associated proteome, offering a deeper understanding of the specific expression patterns of these proteins. These results may be useful for understanding compositional differences among regional products and may inform future studies on the characterization and quality evaluation of red yeast rice-derived EV-enriched fractions.

At the same time, the implications of the present findings should be interpreted cautiously. One important methodological limitation of the present study is that, when applied to a complex fermented-food matrix such as red yeast rice, the isolation procedure is more appropriately interpreted as yielding an EV-enriched fraction rather than a population of unequivocally purified EVs. Although vesicle-like particles were observed by TEM and the particle-size distribution was consistent with nanoscale vesicles, the co-isolation of other extracellular or matrix-derived nanostructures cannot be fully excluded. Such materials may include protein aggregates, membrane fragments, ribonucleoprotein complexes, and other non-vesicular nanoparticles. In addition, although protein concentrations were measured for the EV-enriched fractions, the present study did not include systematic quantification of co-isolated lipids, polysaccharides, or other matrix-derived components. As a result, the overall biochemical composition of the isolated material remains incompletely defined. Accordingly, the proteomic dataset presented here should be interpreted as representing proteins detected in EV-enriched fractions, rather than as definitive evidence that all identified proteins were bona fide vesicular cargo. This study therefore provides compositional and bioinformatic evidence only and does not evaluate gastrointestinal stability, uptake, bioavailability, recipient-cell responses, animal outcomes, or human health effects. Our data do not demonstrate nutritional, therapeutic, regenerative, or health-promoting functions of REVs after consumption. Rather, the present work establishes a foundation for future studies aimed at determining the biological relevance of these vesicle-associated components in appropriate in vitro and in vivo models.

Current research on extracellular vesicles from fermented foods has focused mainly on their isolation, physicochemical characterization, and molecular composition, whereas the mechanisms underlying EV biogenesis, cargo loading, release, and biological activity remain insufficiently understood. Future studies incorporating additional purification procedures, vesicle-specific validation methods, and functional assays will be necessary to refine the molecular definition of red yeast rice-derived EVs and to determine whether the proteins identified here have measurable effects beyond the fermentation environment. In this context, advances in multi-omics approaches and computational analyses may help clarify the molecular relationships among microbial communities, extracellular vesicles, and fermentation processes. Such efforts will be necessary before any conclusions can be drawn regarding possible applications of food-derived vesicles beyond their compositional characterization.

## Figures and Tables

**Table 1:** Physicochemical characterization of EV-enriched fractions isolated from red yeast rice samples.

Sample ID	Protein Concentration (μg/mL)	Particle Concentration (Particles/mL)	Mean Particle Size (nm)	Particle-to-Protein Ratio (Particles/μg)
1	106.0	2.3 × 10^11^	153.0	2.2 × 10^9^
2	137.0	3.8 × 10^11^	141.6	2.8 × 10^9^
3	72.0	1.0 × 10^11^	118.9	1.4 × 10^9^
4	178.0	3.6 × 10^11^	129.8	2.0 × 10^9^
5	100.0	1.7 × 10^11^	141.2	1.7 × 10^9^
6	151.0	6.9 × 10^11^	134.5	4.6 × 10^9^
7	254.0	1.3 × 10^12^	131.3	5.1 × 10^9^
8	77.6	2.2 × 10^11^	130.5	2.8 × 10^9^
9	226.0	1.0 × 10^12^	155.2	4.4 × 10^9^
10	265.0	7.8 × 10^11^	127.1	2.9 × 10^9^
11	339.0	7.1 × 10^11^	140.5	2.1 × 10^9^
12	149.0	1.2 × 10^11^	128.0	8.1 × 10^8^
13	116.0	2.5 × 10^11^	132.0	2.2 × 10^9^
14	182.0	5.1 × 10^11^	146.3	2.8 × 10^9^
15	236.0	5.6 × 10^11^	145.5	2.4 × 10^9^
16	111.0	9.7 × 10^10^	113.7	8.7 × 10^8^
17	156.0	3.6 × 10^11^	121.2	2.3 × 10^9^
18	267.0	4.5 × 10^11^	155.8	1.7 × 10^9^
19	169.0	2.9 × 10^11^	110.8	1.7 × 10^9^
20	188.0	3.4 × 10^11^	133.2	1.8 × 10^9^
21	132.0	1.7 × 10^11^	120.5	1.3 × 10^9^
22	312.0	6.6 × 10^11^	132.6	2.1 × 10^9^
23	176.0	1.2 × 10^12^	180.5	6.8 × 10^9^
24	109.0	9.4 × 10^10^	128.8	8.6 × 10^8^
Mean ± SD	172.1 ± 72.0	4.60 ± 3.44 × 10^11^	135.5 ± 15.5	2.47 ± 1.52 × 10^9^
Range	72.0–339.0	9.4 × 10^10^–1.3 × 10^12^	110.8–180.5	8.1 × 10^8^–6.8 × 10^9^

Note: Particle concentration and mean particle size were determined by NTA. Protein concentration was measured for each EV-enriched fraction after isolation. The particle-to-protein ratio was calculated as the number of particles per microgram of protein and is provided as an additional physicochemical descriptor of the EV-enriched fractions rather than a definitive purity metric.

## Data Availability

The original data presented in the study are openly available in Mendeley Data at doi: https://doi.org/10.17632/37y46zmvkp.1.

## Supplementary Materials

The following supporting information can be downloaded at https://www.mdpi.com/article/10.3390/biom16101425/s1. Data S1. Original ZetaView NTA report for the representative EV-enriched fraction sample 1. Data S2. Summary of 11-position NTA measurements for representative sample 1. Data S3. Raw ZetaView NTA output for the representative EV-enriched fraction sample 1. Figure S1: KEGG pathway analysis of differentially expressed proteins between (A) FJ and GX, (B) FJ and ZJ, and (C) GX and ZJ; Figure S2: KEGG pathway enrichment analysis for (A) *Pseudomonadota* and (B) *Ascomycota*; Table S1: Red yeast rice source information; Table S2: All identified proteins; Table S3: Microbial species annotation of metaproteomic data based on the UniProt database; Table S4: GO biological process enrichment analysis for 273 annotated proteins; Table S5: GO cellular component enrichment analysis for 273 annotated proteins; Table S6: GO molecular function enrichment analysis for 273 annotated proteins; Table S7: KEGG enrichment analysis for 273 annotated proteins; Table S8: Differentially expressed proteins; Table S9: Abundance analysis of dominant prokaryotic and eukaryotic taxa and REV proteins across three regions; Table S10: Taxonomic analysis of REV proteins at the phylum level; Table S11: Taxonomic analysis of REV proteins at the class level; Table S12: Taxonomic analysis of REV proteins at the order level; Table S13: Taxonomic analysis of REV proteins at the genus level; Table S14: Mean protein quantification values of microbial genera; and Table S15: Phylum-specific KEGG functions and pathways.
