# Supplementary material for: Proteomic Characterization of Microbiota-Derived Extracellular Vesicle-Enriched Fractions Detected in Red Yeast Rice
Source: Biomolecules. 2026 Oct 1;16(10):1425. doi: 10.3390/biom16101425 (PMC13702247; doi:10.3390/biom16101425)
Supplement: Supplementary file 1 [file biomolecules-16-01425-s001.zip › biomolecules-4571542-supplementary - New/Supplemental files/Supplemental files.pdf]

Supplemental Information for

Proteomic Characterization of Microbiota-Derived Extracellular Vesicle-Enriched Fractions Detected in Red Yeast Rice

Wei Qi*, Xuan-Xi Peng, Pei-Yan Song, San-Tong Xiao, Wen-Zhong Hu*

College of Life Science, Zhuhai College of Science and Technology, Zhuhai 519041, Guang Dong, P. R. China

This file includes:

Supplementary data (S1-S3)

Supplementary tables (Table S1-S15)

Supplementary figures (Figures S1-S2)

Supplementary data legends

Data S1. Original ZetaView NTA report for the representative EV-enriched fraction sample 1.

Data S2. Summary of 11-position NTA measurements for representative sample 1.

Data S3. Raw ZetaView NTA output for the representative EV-enriched fraction

sample 1.

Supplementary table legends

Table S1. Red yeast rice source information.

Table S2. All identified proteins.

Table S3. Microbial species annotation of metaproteomic data based on the UniProt database

Table S4. GO biological process enrichment analysis for 273 annotated proteins.

Table S5. GO cellular component enrichment analysis for 273 annotated proteins.

Table S6. GO molecular function enrichment analysis for 273 annotated proteins.

Table S7. KEGG enrichment analysis for 273 annotated proteins.

Table S8. Differentially expressed proteins.

Table S9. Abundance analysis of dominant prokaryotic and eukaryotic taxa and REV proteins across three regions.

Table S10. Taxonomic analysis of REV proteins at the phylum level.

Table S11. Taxonomic analysis of REV proteins at the class level.

Table S12. Taxonomic analysis of REV proteins at the order level.

Table S13. Taxonomic analysis of REV proteins at the genus level.

Table S14. Mean protein quantification values of microbial genera

Table S15. Phylum-specific KEGG functions and pathways.

Supplementary figure legends

Figure S1 KEGG pathway analysis of differentially expressed proteins between (A) FJ and GX, (B) FJ and ZJ, and (C) GX and ZJ.

Figure S2 KEGG pathway enrichment analysis for (A) *Pseudomonadota* and (B) *Ascomycota*.
