# Supplementary material for: Proteomic Characterization of Microbiota-Derived Extracellular Vesicle-Enriched Fractions Detected in Red Yeast Rice
Source: Biomolecules. 2026 Oct 1;16(10):1425. doi: 10.3390/biom16101425 (PMC13702247; doi:10.3390/biom16101425)
Supplement: Supplementary file 1 [file biomolecules-16-01425-s001.zip › biomolecules-4571542-supplementary - New/Supplemental files/Supplementary data S1. Original ZetaView NTA report for the representative EV-enriched fraction sample 1.pdf]

Electrophoresis & Brownian Motion
Video Analysis
Laser Scattering Microscopy

www.particle-metrix.de

Video Operator: ZetaUser

Operator (Report): ZetaUser

Sample Parameters
Sample Name: HQM-1
Comment: EV/ 488nm laser
Sample Remarks0:
Sample Remarks1:
Sample Remarks2:
Electrolyte: PBS
Temperature: 25.61 °C sensed
pH 7.0 entered

Instrument Parameters
Laser Wavelength: 488 nm
Filter Wavelength: Scatter

SOP: EV-exosome.
Size Distribution 1 Cycle 11 Positions, 1 Removed for Analysis

Result (sizes in nm)

	Number	Concentration	Volume
Median (X50)	140.6	140.6	208.5
Span	55.2	55.1	75.5

Concentration: 5.8E+7 Particles / mL
Dilution Factor: 4000
Original Concentration: 2.3E+11 Particles / mL

Quality
Average Counted Particles per Frame: 145
Number of Traced Particles: 1235

Analysis Parameters
Max Area: 1000, Min Area: 10, Min Brightness: 30

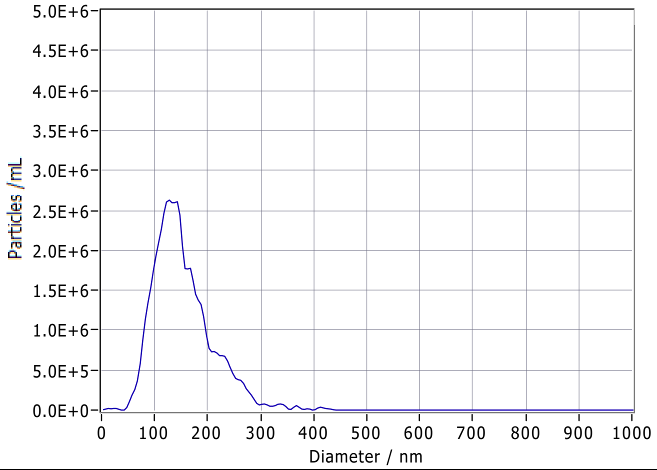

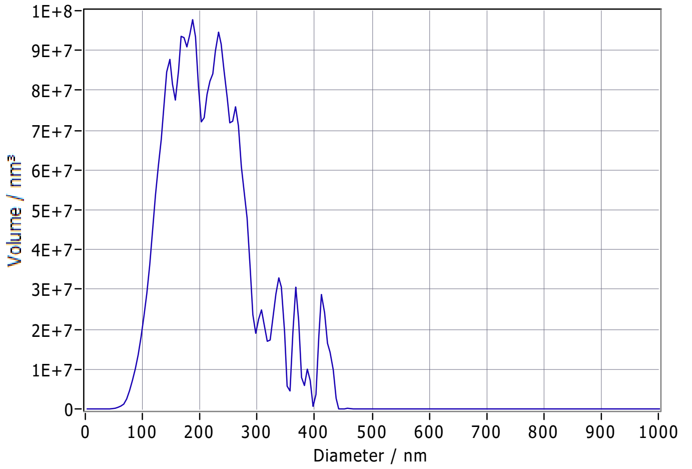

Peak Analysis (Concentration)

Diameter / nm	Particles/mL	FWHM / nm	Percentage
133.8	2.6E+6	100.9	100.0

X Values (all sizes are given in nm)

	Number	Concentration	Volume
X10	90.2	90.2	129.5
X50	140.6	140.6	208.5
X90	227.6	227.6	329.0
Span	1.0	1.0	1.0
Mean	153.0	153.0	219.7
StdDev	55.2	55.1	75.5

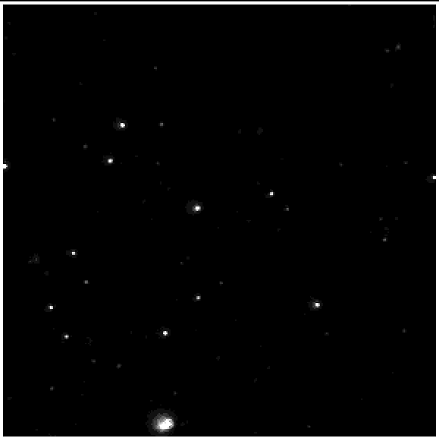

Comment

(Signature)

Analyzed Video: Z:\Tiffany\20240912\HQM-1\20240912_0050_HQM-1_size_488.avi

ZetaVIEW S/N 21-734, Software ZetaView (version 8.05.14 SP7)

Experiment: 2024-09-12 14:21, Report: 2024-09-12 14:22
